# Molecular mechanisms of CRISPR-mediated microbial immunity

**DOI:** 10.1007/s00018-013-1438-6

**Published:** 2013-08-20

**Authors:** Giedrius Gasiunas, Tomas Sinkunas, Virginijus Siksnys

**Affiliations:** Institute of Biotechnology, Vilnius University, Graiciuno 8, 02241 Vilnius, Lithuania

**Keywords:** Phage-resistance, CRISPR, Interference, Cascade, Cas3, Cas9

## Abstract

Bacteriophages (phages) infect bacteria in order to replicate and burst out of the host, killing the cell, when reproduction is completed. Thus, from a bacterial perspective, phages pose a persistent lethal threat to bacterial populations. Not surprisingly, bacteria evolved multiple defense barriers to interfere with nearly every step of phage life cycles. Phages respond to this selection pressure by counter-evolving their genomes to evade bacterial resistance. The antagonistic interaction between bacteria and rapidly diversifying viruses promotes the evolution and dissemination of bacteriophage-resistance mechanisms in bacteria. Recently, an adaptive microbial immune system, named clustered regularly interspaced short palindromic repeats (CRISPR) and which provides acquired immunity against viruses and plasmids, has been identified. Unlike the restriction–modification anti-phage barrier that subjects to cleavage any foreign DNA lacking a protective methyl-tag in the target site, the CRISPR–Cas systems are invader-specific, adaptive, and heritable. In this review, we focus on the molecular mechanisms of interference/immunity provided by different CRISPR–Cas systems.

**Electronic supplementary material:**

The online version of this article (doi:10.1007/s00018-013-1438-6) contains supplementary material, which is available to authorized users.

## Introduction

Bacteriophages (or viruses of bacteria) are the most abundant organisms in the biosphere. They infect bacteria in order to reproduce and usually kill the host cell when replication is completed. To evade this deadly threat, bacteria evolved multiple phage-resistance mechanisms (defence barriers) that interfere with nearly every step of phage life cycles [1, 2]. Bacteria, for example, mutate receptors to interfere with virus attachment to the cell surface, employ restriction enzymes to destroy viral DNA if it enters the cell, or even commit altruistic suicide to prevent productive virus propagation in the bacteria population. In general, these anti-phage defence barriers often protect bacteria from other invasive DNA molecules like plasmids and other integrative and conjugative elements [1, 2].

Phages overcome bacteria resistance by counter-evolving their genomes. Co-evolution of the T4 phage and *Escherichia coli* restriction–modification system serves as a classical example of defence and counter-attack interactions between phages and their bacterial hosts [3, 4]. The constant arms race ongoing between bacteria and viruses promotes the evolution and dissemination of bacterial bacteriophage-resistance mechanisms [5]. Not surprisingly, a large part of the bacteria genome is occupied by the genes encoding various antiviral defence systems [5]. Some defence barriers, like restriction–modification systems, are able to discriminate “self” versus “non-self” DNA, and in this respect function as a primitive innate immune system which confers resistance against invasive nucleic acids.

**Fig. 1:**
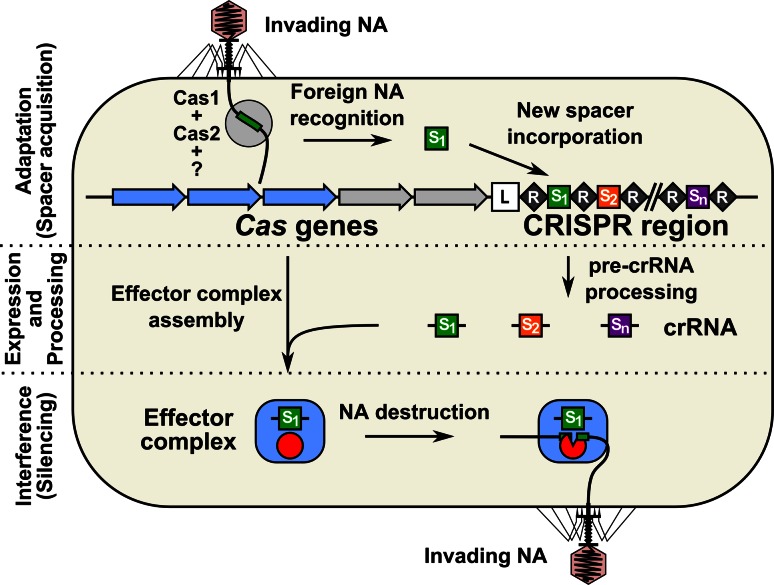
The CRISPR–Cas adaptive microbial immune system confers acquired resistance against invading nucleic acids. CRISPR array consists of short partially palindromic repeats (*black diamonds*) interspaced by unique DNA sequences called spacers (*colored squares*). *Cas* genes (*arrows*) are encoded in the vicinity of the CRISPR array. The CRISPR–Cas mechanism is arbitrarily divided into three main stages: (1) adaptation or spacer acquisition, (2) expression and processing (crRNA generation), and (3) interference or silencing. During adaptation, Cas proteins recognize invasive nucleic acid (*NA*) and integrate short pieces of foreign DNA into the CRISPR region as new spacers. Spacers are inserted at the leader (*L*) proximal end followed by duplication of the repeat. From the perspective of the microbial immune system, the adaptation step is analogous to the immunization of bacteria by an invasive nucleic acid and memorization of the invader. In the expression and processing stage, the CRISPR repeat-spacer array is transcribed into a long primary RNA transcript (*pre-crRNA*) that is further processed into a set of small crRNAs, containing a conserved repeat fragment and a variable spacer sequence (guide) complementary to the invading nucleic acid. crRNAs further combine with Cas proteins into an effector complex. In the interference or silencing stage, the effector complex recognizes the target sequence in the invasive nucleic acid by base pairing and induces sequence-specific cleavage, thereby preventing proliferation and propagation of foreign genetic elements. From the perspective of the microbial immune system, the expression/interference step would be analogous to the immune response of a “vaccinated” host against invasive nucleic acid

Recently, an adaptive microbial immune system, named clustered regularly interspaced short palindromic repeats (CRISPR) and which provides acquired immunity against viruses and plasmids, has been identified. It consists of an array of highly conserved short DNA repeat (R) sequences (typically 21–48 bp long), which are interspaced by stretches of variable sequence called spacers (S) (typically, between 26 and 72 bp) (Fig. 1). The spacer sequences generally originate from phage or plasmid DNA [6, 7]. A set of *cas* (CRISPR-associated) genes is typically located in the vicinity of the repeat-spacer array [8, 9]. The sequence and length of repeats are conserved within the specific CRISPR locus, but greatly diverge between different CRISPR systems [10, 11]. The number of repeat-spacer units in one CRISPR loci varies from 2 to 249 [11, 12]. Multiple CRISPR–Cas systems might be present in the same genome; the record holder is *Methanocaldococcus jannaschii,* which contains 18 CRISPR loci [11]. CRISPR–Cas systems are widespread, and are found, as of May 7, 2013, in 48 % of bacteria (in 1,025 species from 2,151 sequenced) and more than 85 % of archaea (in 123 species from 145 sequenced) genomes [10].

The CRISPR–Cas mechanism is arbitrarily divided into three main steps: (1) adaptation or spacer acquisition, (2) expression and processing (crRNA generation), and (3) interference or silencing (Fig. 1). During adaptation, the Cas proteins recognize invasive DNA (bacteriophage or plasmid DNA) and integrate short pieces of the foreign DNA into the CRISPR region as new spacers [13–20]. From an immunological point of view, the adaptation step is analogous to the immunization of bacteria by an invasive nucleic acid and memorization of the invader. In this case, acquisition of a new trait by bacteria occurs by a horizontal transfer and follows a Lamarckian rather than a Darwinian mechanism [21]. Next, the CRISPR repeat-spacer array is transcribed into a long primary RNA transcript that is further processed into a set of small CRISPR RNAs (crRNAs), containing a conserved repeat fragment and a variable spacer sequence (guide) complementary to the invading nucleic acid [22–24]. crRNAs further combine with Cas proteins into an effector complex, which recognizes the target sequence in the invasive nucleic acid by base pairing to the complementary strand of double-stranded DNA [25] or single-stranded RNA [24, 26], and induces sequence-specific cleavage [14], thereby preventing proliferation and propagation of foreign genetic elements. Again, from an immunological point of view, the expression/interference step would be analogous to the immune response of a “vaccinated” host against invasive nucleic acid. Thus, in contrast to other bacteriophage-resistance mechanisms, the CRISPR–Cas functions as invader-specific, adaptive and heritable microbial immune system that confers acquired resistance against viruses and plasmids.

CRISPR–Cas systems have been categorized into three main types, based on core elements content and sequences [27]. More than one CRISPR–Cas system type is usually found in one organism, suggesting that these systems are compatible and could share functional components [27, 28]. Initially, four distinct genes encoding conserved Cas proteins were identified and named *cas1*–*4* [8], but subsequent bioinformatic analyses have shown that Cas proteins are much more diverse. According to a current view, *cas* genes encode 65 sets of orthologous Cas proteins, which initially were classified into 45 different families but later cut to 25 families by applying more stringent classification criteria [9, 27, 29]. Only the *cas1* and *cas2* genes seem to be universal and found in the majority of CRISPR–Cas systems. Each type is specified by the so-called signature protein, which is conserved in a particular type; accordingly, Cas3 in Type I, Cas9 in Type II, and Cas10 in Type III.

The mechanisms of the adaption/immunization step which include spacer selection and acquisition are still enigmatic. Cas1 and Cas2 proteins are involved in this process [15]; however molecular details remain obscure. The mechanisms underlying crRNA generation have been established for a number of CRISPR–Cas systems and are reviewed elsewhere [28, 30–38]. Here, we focus on the interference/immunity mechanisms employed by CRISPR–Cas systems of different types.

## DNA interference in Type I CRISPR–Cas systems

Type I systems are subdivided into six subtypes that differ by the number and arrangement of *cas* genes [27]. CRISPR–Cas locus of I-A subtype, as exemplified by *Sulfolobus solfataricus,* is comprised of 12 *cas* genes, while subtype I-F in *Pseudomonas aeruginosa* contains only 6 *cas* genes (Online Resource Fig. S1). Despite the differences, all Type I systems encode a hallmark Cas3 protein alongside a universally conserved Cas1 protein [27]. Type I CRISPR-mediated mechanisms of adaptive immunity have been explored for the six model organisms (Table 1). Two of them (*E. coli* and *Streptococcus thermophilus*) belong to the subtype I-E, while the other four are of I-A (*S. solfataricus*), I-B (*Haloferax volcanii*), I-C (*Bacillus halodurans*), and I-F (*P. aeruginosa*) subtypes, respectively. Although the repeat length and sequences in the Type I CRISPR array vary between the model systems, nucleotide sequences within a repeat are partially palindromic, except for subtype I-A (Table 1). In the Type I systems, repeat-spacer arrays are transcribed into a precursor crRNA (pre-crRNA) where a palindromic sequence of the repeat forms a hairpin, which is recognized and processed by Cas6 or Cas5d endoribonucleases [22, 39] to generate a mature crRNA. crRNA is then incorporated into a large multisubunit RNP complex, which together with Cas3 protein induce silencing of invasive DNA [28, 30–38].

**Table 1:** Features of the Type I CRISPR–Cas systems and effector complexes

Organism	Complex acronym	Sub-type	Repeat length, nt	Repeat sequence^a^	Spacer length, nt	PAM^b,c^ (5′–3′)	crRNA length, nt	Complex composition	Ref.
*Sulfolobus solfataricus*	Ss-Cascade (aCascade)	I-A	24-25	5′-GATAATCTCTT ATAGAATTGAAAG-3′^d^	38–44	nd	60–70	Cas7, Cas5, Csa5, Cas6	[61]
*Haloferax volcanii* ^e^		I-B	30	5′-GCTTCAACCCCACAAG GGTTCGTCTGAAAC-3′	34–39	TTC, ACT, TAA, TAT, TAG, CAC	64–69	nd	[62, 63]
*Bacillus halodurans*	Bh-Cascade	I-C	32	5′-GTCGCACTCTACATGA GTGCGTGGATTGAAAT-3′	33–36	nd	65^f^	Cas8c_1_:Cas7_6_:Cas5d_2_	[39]
*Escherichia coli* K-12	Ec-Cascade	I-E	29 (28)^h^	5′-(G)AGTTCCCCGCGCC AGCGGGGATAAACCG-3′^h^	32 (33)^h^	AWG (AW)^h^	61	Cse1_1_:Cse2_2_:Cas7_6_:Cas5_1_:Cas6e_1_	[25]
*Streptococcus thermophilus* DGCC7710	St-Cascade	I-E	28	5′-GTTTTTCCCGCACAC GCGGGGGTGATCC-3′	33	W	61	Cse1, Cse2, Cas7, Cas5, Cas6e	[55]
*Pseudomonas aeruginosa* PA14	Pa-Cascade	I-F	28	5′-GTTCACTGCCGTA TAGGCAGCTAAGAAA-3′	32	GG	60	Csy1_1_:Csy2_1_:Csy3_6_:Cas6f_1_	[18, 58]

^a^Palindromic sequences in the repeat are underlined

^b^PAM sequences were determined experimentally in vivo or in vitro

^c^In Type I systems, the PAM sequence is localized upstream of a protospacer; the protospacer is defined as sequence identical to the spacer

^d^Repeats are non-palindromic

^e^Cascade complex has not been isolated or characterized

^f^Predicted crRNA length

^g^Effector complex stoichiometry implied from molecular mass data and analogy to I-E Cascade

^h^According Goren et al. [45], in the *E. coli* I-E CRISPR–Cas system, the first G nucleotide of the repeat belongs to the spacer, therefore the boundaries of the repeat, spacer, and PAM might differ

### Subtype I-E

Most of our knowledge on the mechanism of DNA interference in the I-E subtype comes from the studies of two model systems, namely *E. coli* K-12 CRISPR and *Streptococcus thermophilus* DGCC7710 CRISPR4 (Table 1).

#### *E. coli* CRISPR–Cas system

Pioneering studies of the *E. coli* CRISPR system in the van der Oost laboratory provided a paradigm for DNA silencing in Type I systems [23]. The *E. coli* CRISPR–Cas system encodes eight Cas proteins downstream of the CRISPR region (Online Resource Fig. S1) and five Cas proteins assemble into an effector complex that binds crRNA into a RNP complex called CRISPR-associated complex for antiviral defence (Cascade) [23]. Ec-Cascade is a 405-kDa complex comprised of five Cas proteins and crRNA with the following stoichiometry: (Cse1)_1_:(Cse2)_2_:(Cas7)_6_:(Cas5)_1_:(Cas6e)_1_:(crRNA)_1_ (Table 1) [23, 25, 40]. The mature 61-nt crRNA [25] identified in the Ec-Cascade is comprised of a 32-nt spacer flanked by 8-nt 5′- and 21-nt 3′-handles resulting from the pre-crRNA cleavage within repeat stems by the Cas6e endoribonuclease [41, 42]. Ec-Cascade using crRNA as a guide locates the target DNA in a process that has yet to be defined, and binds to the complimentary DNA strand creating an R-loop, if a short protospacer adjacent motif (PAM) [25] is present in the vicinity of matching protospacer. Initially, bioinformatic analysis identified the 5′-AWG-3′ PAM [43] that was required for Ec-Cascade binding and subsequent DNA interference [44]. On the other hand, experimental analysis of CRISPR repeat boundaries in *E. coli* suggests a dinucleotide 5′-AW-3′ as PAM, arguing that the last G nucleotide belongs to the protospacer sequence [45]. The molecular machinery of new spacer integration in *E. coli* recognizes the PAM sequence, of which 2 nt are outside a protospacer and the third G nucleotide becomes an integral part of a protospacer [45]. The PAM definition in the *E. coli* system still remains controversial since the spacer integration analysis in a non-laboratory *E. coli* strain identifies the AWG sequence as PAM [46].

Low resolution electron microscopy of the Ec-Cascade reveals a sea-horse shape [25], which was later confirmed by sub-nanometer resolution structures [40]. In the Ec-Cascade, six copies of Cas7 assemble into a helical structure that provides a binding platform for crRNA and acts as a scaffold physically linking other Cas proteins. Both 3′- and 5′-handles of crRNA originating from the conserved repeat region are anchored by specific interactions with Cas6e ribonuclease and 5′-proximal Cas7/Cse1 subunits, respectively. On the other hand, the Cse2 dimer joining to the Cas7 oligomer creates an extended binding surface for the 3′-half of the crRNA spacer [40], and displays crRNA on the surface of a helical structure made by the Cas7 oligomer. Such binding mode protects crRNA from degradation but maintains base pairing potential with a complementary DNA target [28]. Ec-Cascade binding to the target site culminating in R-loop formation is a complicated process that involves a number of stages including recognition of an alien nucleic acid, PAM localization, and crRNA hybridization to the matching DNA strand. Molecular details for different stages remain to be established; however, key players are already emerging. The Cse1 subunit is presumably involved in PAM recognition by Cascade [47] and, at the same time, serves as a docking site for Cas3 nuclease–helicase [48]. Furthermore, only seven nucleotides proximal to the PAM in the target DNA seem to be crucial for initiation of Ec-Cascade binding, suggesting a similar role to the “seed” sequences in microRNA’s [49–51]. The crRNA “seed” sequence in *E. coli* may play a role in the initial scanning of invader DNA for a perfect match before base pairing of the full-length spacer can occur [44]. The initial DNA strand opening may be triggered by distortion occurring upon Ec-Cascade binding to the PAM sequence and is promoted by a negative supercoiling [48, 52]. The role of DNA topology on Ec-Cascade binding remains to be systematically investigated.

#### *S. thermophilus* CRISPR4–Cas system

The *S. thermophilus* DGCC7710 strain contains four different CRISPR systems which belong to three different Types [31] (Online Resource Fig. S2). Functional activity has been demonstrated in vivo for Type II-A CRISPR1 [13] and CRISPR3 [54] systems; however, neither spacer acquisition nor interference activity has so far been reported for the CRISPR2 and CRISPR4 systems, which belong to the subtypes III-A and I-E, respectively. The St-CRISPR4–Cas of *S. thermophilus* DGCC7710 is orthologous to the I-E CRISPR–Cas system of *E. coli* discussed above [31, 53]. In the St-CRISPR4–Cas system, five *cas* genes are arranged into a cluster (cse1–cse2–cas7–cas5–cas6e) (Online Resource Fig. S2) analogous to the *E. coli cas* genes, suggesting that corresponding Cas proteins may assemble into a homologous St-Cascade complex. Indeed, cloning and expression of the *S. thermophilus* CRISPR4 system in an *E. coli* strain lacking *cas* genes, enabled isolation and characterization of the *S. thermophilus* Cascade (St-Cascade), which consists of five Cas proteins orthologous to the Ec-Cascade [55]. The stoichiometry of the St-Cascade was not directly determined; however, indirect data suggest that, similar to the Ec-Cascade [25], the Cas7 protein is the most abundant protein in St-Cascade. Repeat sequences of *E. coli* and St-CRISPR4 systems differ by 8 nt, however the fragment corresponding to the GC rich hairpin stem in *Ec*-crRNA is conserved. Furthermore, the 61-nt length of crRNA co-purified with St-Cascade is consistent with a conserved cleavage position at the 21st nt of the repeat sequence [55] and implies a conserved mechanism of pre-crRNA processing by Cas6e ribonuclease. In the *S. thermophilus* CRISPR4 system, the mature crRNAs is comprised of a 7-nt 5′-handle, a 33-nt spacer, and a 21-nt 3′-handle (Table 1). It is likely that the St-Cascade complex will be arranged into a similar sea-horse-like structure as established for Ec-Cascade [25, 40].

St-Cascade binding to the matching sequence in the target DNA requires a PAM sequence located in the vicinity of a protospacer. In the St-CRISPR4 system, the PAM predicted by in silico analysis of the matching protospacer sequences in *S. thermophilus* phages is an AA dinucleotide located immediately upstream of the protospacer. Surprisingly, the PAM identified in the in vitro binding assay is extremely promiscuous and limited to a single A(−1) or T(−1) nucleotide [55]. Of note is that in vitro binding affinities of the Ec-Cascade and St-Cascade to the matching DNA sequences containing optimal PAMs differ by at least two orders of magnitude [48, 55]. Taken together, these data demonstrate that (1) orthologous *E. coli* and St-CRISPR4 systems show different PAM dependencies, and (2) the requirements for the PAM stringency in St-CRISPR4 may be different for the spacer acquisition and interference steps [16, 56].

### Subtype I-F

The CRISPR–Cas system of *P. aeruginosa* encodes six *cas* genes (Online Resource Fig. S1). Biochemical and structural evidence indicate that four Cas proteins and crRNA assemble into a 350-kDa RNP complex (Pa-Cascade) of (Csy1)_1_:(Csy2)_1_:(Csy3)_6_:(Cas6f)_1_:(crRNA)_1_ stoichiometry. The complex contains 60 nt of mature crRNA comprised of a 32-nt spacer flanked by 8-nt 5′- and 20-nt 3′-handles which result from the pre-crRNA cleavage by the Cas6f endoribonuclease within a repeat stem [47, 57]. Like Ec-Cascade, Pa-Cascade binds to DNA targets containing crRNA matching sequences [58]. According to the phage challenge assay, the GG PAM is required for a new spacer acquisition by the CRISPR–Cas system of *P. aeruginosa* (Table 1). Isothermal titration calorimetry analysis shows that Pa-Cascade binding is enthalpically driven and governed by a “seed” sequence [58], similar to Ec-Cascade [40, 44]. Moreover, all nucleotides in PAM and seed sequences are important for protection from phage infection [59].

Structural analysis of the Pa-Cascade by native mass spectrometry, electron microscopy, and small-angle X-ray scattering reveals a crescent-shaped particle [58]. In general, the overall shape and stoichiometry of the Csy effector complex are reminiscent of those for the Ec-Cascade. The Pa-Cascade, however, lacks the large extension which is formed by Cse1 protein in Ec-Cascade [58]. Since Cse1 is presumably involved in PAM recognition and target DNA selection [60], the mechanism of target localization and binding by Ec-Cascade and Pa-Cascade may be different.

### Subtype I-A

CRISPR–Cas systems of subtype I-A are more complex compared to I-E or I-F [27]. Three Type I-A CRISPR clusters are identified in the *S. solfataricus* genome (Online Resource Fig. S1). In the largest Type I-A cluster comprised of 12 genes, the RNP complex pulled-down using a strep-tagged Cas7 (SSO1442) protein variant consists of crRNA and the Cas5 (SSO1441) protein. It was called aCascade (for archaeal Cascade) [61]; however, throughout this review, we use the Ss-Cascade acronym to be consistent with acronyms for other complexes (Table 1). Minor amounts of Csa5 (SSO1443) and Cas6 (SSO1437) proteins co-purified with Ss-Cascade, suggesting weak interactions or transient complexes. In contrast to the I-E and I-F CRISPR–Cas systems, where the spacer length is fixed, the spacer in the I-A system varies from 38 to 44 nt. Consequently, the crRNA co-purifying with Ss-Cascade is between 60 and 70 nt, and contains an 8-nt 5′-tag (handle) and a 16–17-nt 3′-tag (handle) resulting from pre-crRNA cleavage by Cas6 in the unstructured repeat region [61]. According to electron microscopy data, Cas7 proteins assemble into a helical structure [61] resembling Ec-Cascade.

The crystal structure of Cas7 reveals a crescent-shaped three-domain architecture [61]. The ferredoxin-like domain, which is frequently employed for RNA binding, forms a Cas7 protein core. Two additional domains that are unique for the Type I-A Cas7 are inserted in the conserved ferredoxin-like domain [61]. Although the Cas7 structure provides no hints on the Cas7 oligomerization interface in the Ss-Cascade helical spine, it predicts putative amino acids residues that may interact with crRNA. Consistent with in silico prediction, the alanine replacement of the conserved His160 significantly decreases Cas7 protein affinity to the crRNA [61]. Moreover, Ss-Cascade reconstituted in vitro by mixing recombinant Cas7 and Cas5 proteins with crRNA binds complementary single-stranded DNA; however, a double-stranded DNA target binding remains to be demonstrated [61].

### Subtype I-B

Although the I-B effector complex has yet to be isolated and characterized in vitro, the in vivo experiments in *H. volcanii* provide a first glimpse into molecular details for I-B systems [62, 63]. *H.* *volcanii* encodes eight *cas* genes and three distinct CRISPR repeat-spacer arrays (Online Resource Fig. S1). The 30-nt repeat sequence in a pre-crRNA forms a minimal hairpin stem comprised of three paired nucleotides and cleavage occurs within the stem base to yield an 8-nt 5′-tag (handle) and a 22-nt 3′-tag (handle) in the mature crRNA. Interestingly, the crRNAs generated from the three distinct CRISPR repeat-spacer loci contain different 5′-terminal nucleotides (U, G, or A). Furthermore, the spacer length in the *H. volcanii* I-B system varies between 34 and 39 nt, and therefore the length of the mature crRNA is between 64 and 69 nt [62]). The subtype I-B CRISPR–Cas system of *H. volcanii* provides interference against invading plasmids if PAM sequences (TTC, ACT, TAA, TAT, TAG, and CAC) are located in the vicinity of the protospacer (Table 1) [62, 63].

### Subtype I-C

The *B. halodurans* encodes seven *cas* genes arranged in the I-C cluster (Online Resource Fig. S1). Differently from other Type I systems, I-C does not encode the Cas6 protein required for crRNA maturation [27]. In the I-C CRISPR–Cas system, the Cas5d protein replaces the Cas6 endoribonuclease and cleaves pre-crRNA to generate a mature crRNA [39, 64]. The repeat sequence in *B. halodurans* is partially palindromic and folds into a hairpin structure in the pre-crRNA. However, unlike the Ec-Cas6 endoribonuclease, the Cas5d cleaves at the base of hairpin to yield 11-nt 5′- and 21-nt 3′-tags (handles) originating from the conserved repeat regions [39].

Cas5d recognizes both the hairpin structure of the repeat and a single-stranded RNA fragment at the 3′-end of the repeat. After pre-crRNA processing, one Cas5d subunit remains bound to the 3′-repeat handle, while another subunit presumably interacts with the 8-nt 5′-repeat handle of matured crRNA. Finally, Cas5d together with crRNA, Cas8c (Csd1), and Cas7 (Csd2) proteins assemble into a 400-kDa RNP complex following (Cas8c)_1_:(Cas7)_6_:(Cas5d)_2_:(crRNA)_1_ stoichiometry (where Cas8c is an Cse1 ortholog in I-E) and exhibits spatial architecture similar to Ec-Cascade (Table 1) [39]. Interestingly, when Cas8c, Cas7, and Cas5d proteins are expressed in *E. coli* cells lacking one of Ec-Cascade components (Cse1, Cas7, Cas5, or Cas6e, respectively) but containing endogenous *E. coli* CRISPR loci and Cas3, the silencing by the heterologous Type I-C system is restored, suggesting the possible complementation between different CRISPR-Cas systems [39, 65].

### Cas3: a slicer for DNA in Type I systems

Ec-Cascade binding to the matching sequence in the invading DNA does not trigger silencing; degradation of the foreign DNA requires an accessory Cas3 protein [23]. Cas3 is a signature protein of the Type I systems [27] and typically contains HD phosphohydrolase and Superfamily 2 helicase domains arranged into a single subunit protein; however, sometimes, HD- and helicase-domains are encoded as individual Cas3′ and Cas3″ subunits, respectively [9, 27, 29]. Furthermore, in some CRISPR systems, the single chain Cas3 or separate Cas3 domains are fused to other Cas proteins (Cas2–Cas3, Cas3–Cse1) [27, 48]. Single chain Cas3 variants from four different bacteria/archaea strains and Cas3 domains (subunits) have been purified and biochemically characterized (Table 2; Online Resource Figs. S1, S2).

**Table 2:** Biochemical properties of Cas3 proteins

Organism	Complex acronym	Sub-type	Arrangement	ATPase activity	Helicase activity	Nuclease activity	Other activities	Ref.
*Sulfolobus solfataricus *P2	Ss-Cas3″	I-A	Hel; HD^a^	nd	nd	Endo dsDNA/dsRNA, traces on ssDNA/RNA	–	[69]
*Methanocaldococcus jannaschii*	Mj-Cas3′; Mj-Cas3″	I-A	Hel; HD^b^	+	+	Endo ssDNA/ssRNA, and exo (3′ → 5′)	R-loops, DNA flaps cleavage	[68]
*Methanothermobacter thermautotrophicus*	Mt-Cas3	I-C	HD-Helicase	nd	+	nd	R-loop formation and dissociation	[66]
*Escherichia coli* K12	Ec-Cas3	I-E	HD-Helicase	nd	+	nd	R-loop formation and dissociation	[66, 72]
*Streptococcus thermophilus* DGCC7710	St-Cas3	I-E	HD-Helicase	+	3′ → 5′	Endo ssDNA	–	[53]
*Thermus thermophilus* HB8	Tt-Cas3	I-E	HD^c^-Helicase	nd	nd	Endo ssDNA	–	[67]

^a^Two separate proteins

^b^Two separate proteins; only the HD domain carrying protein was purified and analyzed

^c^Only the HD domain was purified and analyzed

#### Cas3 of *S. thermophilus* DGCC7710

Consistent with in silico predictions [9, 29], biochemical studies of St-Cas3 revealed that the N-terminal HD-domain is a single-stranded DNA nuclease, while the C-terminal helicase domain possesses a single-stranded DNA-stimulated ATPase activity, which is coupled to unwinding of DNA/DNA and RNA/DNA duplexes in the 3′–5′ direction (Table 2) [53]. In vitro reconstitution of the Type I-E system of *S. thermophilus* DGCC7710 provided the first molecular details for the functional interplay between St-Cascade and St-Cas3 [55]. St-Cas3 alone neither binds nor cleaves dsDNA and has no ATPase or helicase activity [53]. To unleash St-Cas3 full catalytic activity, a single-stranded DNA is required and St-Cascade contributes to its formation. St-Cascade binding to the dsDNA guided by crRNA creates an R-loop where the non-target strand of a protospacer is expelled as ssDNA [55]. This displaced DNA strand in the St-Cascade–target DNA complex serves as a platform for the St-Cas3 binding and triggers ATPase and nuclease activities. The nuclease function of Cas3 located in the HD-domain seems to be coupled to ATP hydrolysis in the C-terminal helicase domain. Indeed, if ATP is missing, the single-stranded DNase activity of the HD domain is weak and cleavage is limited to the expelled non-target DNA strand. On the other hand, in the presence of ATP, the HD-nuclease of Cas3 produces multiple cuts beyond the protospacer region in the 3′–5′ direction, presumably due to the unwinding of DNA duplex by the C-terminal helicase. Interestingly, under these conditions, St-Cas3 also cleaves the crRNA-bound DNA strand either because of the strand switch or additional St-Cas3 molecule binding [55]. As a consequence, in the presence of ATP, the interplay between the nuclease and ATPase/helicase activities of St-Cas3 results in the degradation of plasmid DNA. Of note is that, in the presence of ATP, a few nuclease cuts also occur in the DNA strand engaged in the heteroduplex with crRNA, suggesting a remodeling of the St-Cascade complex.

#### Cas3 of *E. coli*

Due to the poor solubility and a tendency to aggregate, the *E. coli* Cas3 protein (Ec-Cas3) can only be expressed and isolated in very limited amounts [48, 53, 66]. It has been shown recently that, in vivo, the Ec-Cas3 protein interacts with the Ec-Cascade complex via the Cse1 protein. Guided by this finding, Westra et al. [48] have engineered an artificial Ec-Cas3–Cse1 protein fusion that was incorporated into the Ec-Cascade effector complex and provided resistance in vivo. Both HD-nuclease and helicase domains of Ec-Cas3 were important for CRISPR-encoded immunity [48]. Moreover, the Ec-Cas3–Cse1 fusion protein in the in vitro-reconstituted Ec-Cascade complex degraded plasmid DNA containing a crRNA matching protospacer sequence [48]. The ATPase/helicase activity was required for the plasmid degradation suggesting that DNA unwinding may be important for the Cas3 function. The unwinding of DNA–RNA duplexes activity was also reported for the WT Cas3 from *E. coli* (Table 2) [66].

#### Cas3 from other Type I systems

The *T. thermophilus* HB8 Cas3 protein (Tt-Cas3) HD domain belonging to the subtype I-E CRISPR locus reveals Ni^2+^-dependent endonuclease activity on a single-stranded DNA [67]. The cleavage pattern of the Tt-Cas3 in the reconstituted effector complex including the Tt-Cascade has yet to be established. The HD domain protein from the *M. jannaschii* (MjCas3′) (subtype I-A) displays Mg^2+^-dependent endo- and 3′ → 5′ exonuclease activity on single-stranded DNA and RNA, as well as on 3′-flaps, splayed arms, and R-loops, which represent the potential intermediates of DNA degradation [68]. The degradation of branched DNA substrates by MjCas3′ is stimulated by the helicase subunit (MjCas3″) and ATP. In contrast to all other biochemically characterized Cas3 proteins or HD domains (Table 2) that act on single-stranded nucleic acids, the HD-type nuclease subunit (Cas3′) of *S. solfataricus* CRISPR–Cas system has been reported to degrade double-stranded DNA and RNA with a preference to G or C bases [69].

### Mechanism of DNA-interference in the Type I systems

**Fig. 2:**
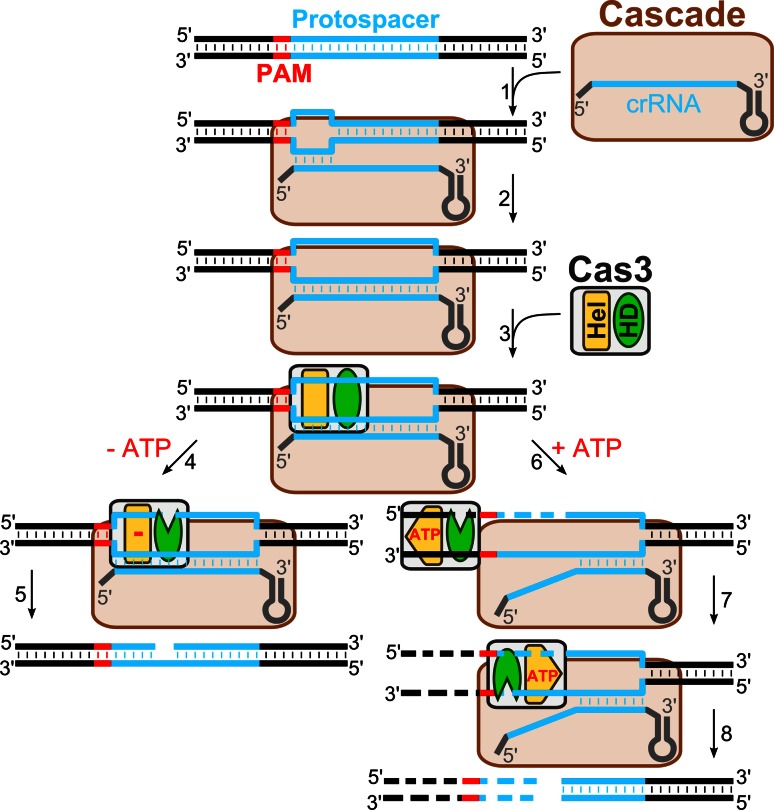
DNA-interference in the Type I CRISPR–Cas systems. Cascade scans DNA for a protospacer sequence and PAM. Once the correct PAM and a short primary hybridization sequence (“seed”) are identified (*1*), the crRNA basepairs with a complementary DNA strand forming R-loop (*2*). Displaced DNA strand of the R-loop serves as landing site for Cas3 (*3*). In the absence of ATP, the Cas3 nuclease domain (*HD*) cleaves a displaced non-target strand within a protospacer (*4*) producing a nicked DNA (*5*). In the presence of ATP, Cas3 remodels the Cascade–DNA complex making both target and non-target strands available for the Cas3 cleavage within a protospacer sequence (*6*). Cas3 further translocates in the 3′ → 5′ direction powered by a helicase domain (*Hel*) whereas the HD domain degrades DNA (*6*; *7*) in a unidirectional manner (*8*)

In summary, biochemical and structural studies of the CRISPR-encoded immunity in the different Type I CRISPR–Cas systems are consistent with a following general mechanism of DNA interference (Fig. 2): (1) crRNA is incorporated into a multisubunit RNP complex (Cascade); the Cascade complex composition and stoichiometry differ between different subtypes but the overall shape of the complex shows a characteristic helical spine similar to the Ec-Cascade, (2) Cascade guided by the crRNA locates the target DNA site and, if the correct PAM sequence is present, binds to the matching DNA strand, creating an R-loop that serves as a loading site for the Cas3 protein, (3) Cas3 binding to the ssDNA triggers ATPase/helicase activity that presumably contributes to Cascade remodelling, making both DNA strands in the protospacer region available for Cas3 cleavage, and (4) after cleaving both DNA strands within the protospacer, Cas3 translocates on the non-target strand in the 3′ → 5′ direction in an ATP-dependent manner and cleaves the translocating strand using its HD-nuclease domain.

### Accessory functions of Cascade and Cas3

The existing experimental evidence suggests that binding of the Cascade surveillance complex marks invasive DNA for destruction, while Cas3 actually acts as a slicer that degrades DNA to provide interference. Recent findings suggest that the CRISPR interference machinery (Cascade and Cas3) may also be important in the spacer acquisition (immunization) stage [16, 19]. Indeed, in the *E. coli* K12 CRISPR system, the integration of new spacers from the infecting phage M13 DNA occurs more often, when crRNA is no longer capable of eliciting defence because the phage harbors an escape mutation in the protospacer (or PAM), but due to the low affinity binding Ec-Cascade is still able to act as a priming site for a new spacer acquisition. The selection of new spacers is largely determined by the priming protospacer orientation and therefore the mechanism is termed priming [19, 70]. A similar mechanism may play a role in the acquisition of new spacers derived from the plasmid [16]. Indeed, when multiple spacers originating from the plasmid are integrated in the *E. coli* CRISPR loci, all spacers target the same strand of DNA, implying that the first acquired spacer directs strand selection for integration of successive spacers [16]. It has been proposed that Cascade–crRNA binding to the matching protospacer sequence determines which strand will be extruded into the R-loop and subjected to degradation in the 3′ → 5′ direction [53, 55]. In this way, the unidirectional DNA degradation by Cas3 may contribute to the selection of a specific DNA strand from which new spacers are subsequently acquired [19]. The molecular mechanism of Cas3 and Cascade in the primed spacer acquisition remains to be established.

In the *Sulfolobus islandicus* CRISPR system, Cas3 is presumably involved in the pre-crRNA maturation [71]. Indeed, in Δcas3′ and Δcas3″ mutants, pre-crRNA processing intermediates accumulate, suggesting that the helicase and nuclease activities of Cas3 are important for the resolution of the processing intermediates and crRNA degradation in *S. islandicus*. Interestingly, Cas3 expression in *E. coli* also triggers cellular processes unrelated to the CRISPR-encoded immunity: in the presence of Cas3, the regulation of ColE1 replicon is impaired, resulting in the higher plasmid copy number [72]. The molecular details of Cas3 in the uncontrolled plasmid replication process have yet to be established, but the helicase domain of Ec-Cas3 seems to be involved. It is possible that the Ec-Cas3 ability to process R-loops without DNA/RNA sequence-specificity and without Cascade may contribute to this process [66].

## DNA interference in Type II CRISPR–Cas systems

In the Type II systems, *cas* genes operon encodes only three or four Cas proteins [27], including the universal Cas1 and Cas2 proteins, as well as Cas4 [73] or Csn2 [74–77], which are involved in the spacer acquisition stage [13, 78]. Type II systems are further subdivided into II-A, II-B, and II-C subtypes [27, 79]. Furthermore, all Type II CRISPR–Cas systems contain a conserved Cas9 signature protein [27]. Hence, the CRISPR-mediated mechanisms of immunity in the Type II systems must be different from those employed by Type I. First, the endoribonucleases (Cas6e or Cas5d) that are involved in the pre-crRNA processing in the Type I systems are missing in the Type II. Surprisingly, in the *Streptococcus pyogenes* II-A CRISPR–Cas system (Online Resource Fig. S1), a trans-encoded small RNA (called a trans-activating CRISPR RNA or tracrRNA) with 24-nt complementarity to the repeat regions of pre-crRNA, contributes to the crRNA maturation in a process that requires host RNase III [80]. The importance of tracrRNA in the crRNA maturation pathway is now directly demonstrated for several Type II systems, and bioinformatic analysis predicts tracrRNA orthologs in most of the Type II systems [79–81]. Second, the Cas3 protein which is involved in the destruction of invading nucleic acids, is missing.

CRISPR-mediated mechanisms of an adaptive immunity for Type II systems have been explored for two model organisms, namely, *S. pyogenes* and *S. thermophilus* DGCC7710 (Table 3). *S. thermophilus* DGCC7710 carries two II-A systems (Online Resource Fig. S2), namely, St-CRISPR1 and St-CRISPR3, and both are active in vivo, e.g., are able to incorporate new spacers upon phage challenge and provide resistance in subsequent rounds of infection [13, 54]. Repeat sequences in the characterized II-A systems are 36 nt in length and partially palindromic (Table 3) [31]. Processing of the pre-crRNA in the presence of tracrRNA and RNase III results in the mature 42-nt crRNA which is considerably shorter in comparison to the crRNA of Type I systems (Table 1). There are two major differences between mature crRNAs’ in II-A and Type I systems. First, crRNA in Type II lacks a 5′-handle and contains an extended 22-nt 3′-handle generated by the RNase III cleavage within the repeat region in the pre-crRNA:tracrRNA duplex. Second, the spacer fragment in the Type II crRNA is shorter, because the 5′-end of the spacer sequence is trimmed to 20 nt by unknown nuclease(s). Consequently, the spacer in the mature crRNA matches only 20 of the 30-nt protospacer sequence in the invading nucleic acid. The non-matching fragment in the protospacer is not important for the CRISPR-mediated immunity; however, shortening of the protospacer sequence to 19 nt or more abrogates CRISPR-mediated plasmid interference [82–84]. Three model systems have been used to study mechanisms of invading nucleic acid destruction by Type II systems.

**Table 3:** Features of the Type II CRISPR–Cas systems and effector complexes

Organism/system	Sub-type	Complex acronym	Repeat length	Repeat sequence	Spacer length	crRNA, nt	tracrRNA	PAM(5′–3′)	Cleavage position	Complex composition	Ref.
*Streptococcus thermophilus* DGCC7710/CRISPR1	II-A^a^		36	5′-GTTTTTGTACTCTCAAGAT TTAAGTAACTGTACAAC-3′	30	~42^b^	+	NNAAGAW	3 nt upstream PAM	nd	[14, 80]
*Streptococcus thermophilus* DGCC7710/CRISPR3	II-A	St-Cas9t	36	5′-GTTTTAGAGCTGTGTTGT TTCGAATGGTTCCAAAAC-3′	30	42	+	NGGNG	3 nt upstream PAM	Cas9–crRNA–tracrRNA	[81, 82]
*Streptococcus pyogenes* SF370	II-A	Sp-Cas9t	36	5′-GTTTTAGAGCTATGCTGT TTTGAATGGTCCCAAAAC-3′	30	42	+	NGG	3 nt upstrem PAM	Cas9–crRNA–tracrRNA	[80, 83]

^a^The Cas9t complex has not been isolated or characterized

^b^The approximate length of crRNA was determined by northern blot [80]

### CRISPR3–Cas of *S. thermophilus* DGCC7710

The St-CRISPR3–Cas system (Online Resource Fig. S2) when transferred into *E*. *coli* confers protection against plasmid transformation and de novo phage infection [78]. The interference against phage and plasmid DNA provided by St-CRISPR3 requires the presence, within the target DNA, of a protospacer sequence complementary to the spacer-derived crRNA, and a conserved PAM sequence, NGGNG, located immediately downstream of the protospacer (Table 3) [43, 54, 85, 86]. In the heterologous system, *cas9* is the sole *cas* gene necessary for the CRISPR-encoded interference [78], suggesting that this protein is involved in crRNA processing and/or crRNA-mediated silencing of invasive DNA.

Cas9 of the *S. thermophilus* CRISPR3–Cas system is a large, multidomain protein comprising 1,409 amino acid residues [78]. It contains two nuclease domains, a RuvC-like nuclease domain near the amino terminus and a HNH-like nuclease domain in the middle of the protein. Mutational analysis has established that interference provided in vivo by Cas9 requires both the RuvC and HNH motifs [78]. The Cas9 protein of the *S. thermophilus* CRISPR3–Cas system co-purifies with a 42-nt crRNA and ~65-nt tracrRNA [81, 82]. Such a ternary complex cleaves both synthetic oligodeoxynucleotide and plasmid DNA bearing a nucleotide sequence complementary to the crRNA, in a PAM-dependent manner. RuvC and HNH active sites of Cas9 are responsible for the cleavage of opposite DNA strands within a protospacer 3 nt away from the PAM sequence. Taken together, our data demonstrate that the Cas9–crRNA–tracrRNA (Cas9t) complex functions as an RNA-guided endonuclease that uses RNA for target site recognition and Cas9 for DNA cleavage.

### CRISPR01–Cas of *S. pyogenes*

The *S. pyogenes* genome contains Sp-CRISPR01–Cas and Sp-CRISPR02–Cas loci that belong to Type II and Type I-C, respectively. The Sp-CRISPR01–Cas (Online Resource Fig. S1) is homologous to the St-CRISPR3–Cas [80]. Sp-Cas9 is responsible for the production of mature crRNAs and interference [80]. Jinek et al. isolated Sp-Cas9 and demonstrated that it cleaves plasmid DNA or an oligonucleotide duplex if target contains a protospacer sequence complementary to the spacer-derived crRNA and a conserved PAM sequence, NGG, located immediately downstream of the protospacer (Table 3). The mature crRNA alone is incapable of directing Sp-Cas9 cleavage and requires accessory tracrRNA to trigger cleavage. Furthermore, a dual-tracrRNA:crRNA engineered as a single RNA chimera supports Cas9-mediated dsDNA cleavage. Sp-Cas9 cleavage occurs in both DNA strands 3 nt away from the PAM sequence. The Cas9 HNH-nuclease domain cleaves the crRNA complementary strand, whereas the Cas9 RuvC-like domain cuts the non-complementary strand.

### CRISPR1–Cas of *S. thermophilus* DGCC7710

The St-CRISPR1–Cas system is similar to that of the St-CRISPR3–Cas in terms of the number and arrangement of associated *cas* genes (Online Resource Fig. S2). The repeat length is identical between the two systems and repeat sequences share 53 % of identity (Table 3). The St-CRISPR1–Cas system provides interference against invading nucleic acids by cleaving foreign DNA carrying a protospacer complementary to the spacer-derived crRNA, and a conserved PAM sequence, NNAAGAW, located immediately downstream of the protospacer [54, 85]. In vivo, the CRISPR1–Cas system specifically cleaves plasmid and bacteriophage double-stranded DNA within the protospacer, 3 nt away from the PAM sequence [14]. The DNA cleavage position determined for the St-CRISPR1–Cas system is identical to that of St-CRISPR3–Cas. Furthermore, in the St-CRISPR1–Cas system, disruption of *cas9* abolishes crRNA-mediated DNA interference in vivo [13, 14]. Therefore, it is likely that DNA cleavage occurs by the Cas9–crRNA complex; however, a detailed mechanism of DNA interference for St-CRISPR3–Cas has yet to be established.

### Mechanism of DNA-interference in the Type II systems

**Fig. 3:**
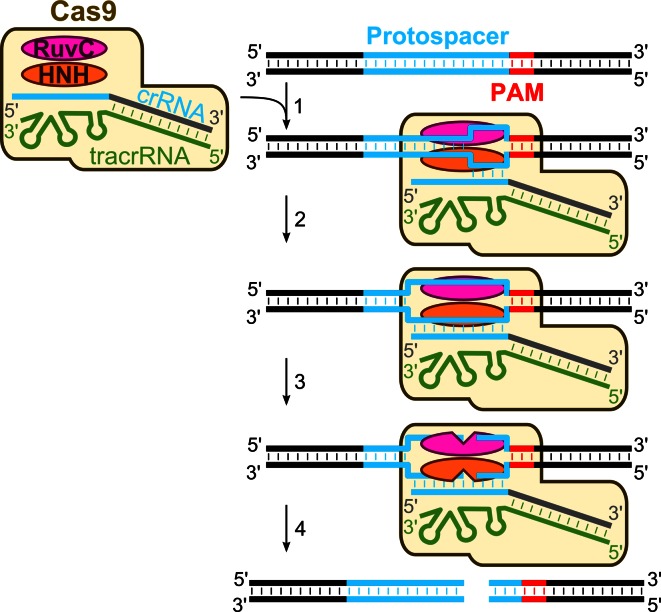
DNA-interference in the Type II CRISPR–Cas systems. The Cas9–crRNA–tracrRNA ternary complex scans DNA for a protospacer sequence and PAM. Once the correct PAM and a short primary hybridization sequence (“seed”) are identified (*1*), the crRNA basepairs with a complementary DNA strand forming R-loop (*2*). Once the R-loop is formed, Cas9 cuts both target and non-target DNA strands using the RuvC and the HNH active sites, respectively (*3*). Cleavage occurs 3 nt before PAM, yielding blunt-end DNA products (*4*)

In summary, genetic and biochemical studies of the CRISPR-encoded immunity in the Type II CRISPR–Cas systems are consistent with the following general mechanism (Fig. 3) of DNA interference. First, the CRISPR repeat region is transcribed into a long primary pre-crRNA which pairs with tracrRNA and undergoes processing by host RNase III to generate a mature crRNA. Cas9 protein contributes to this stage by promoting the formation of the specific crRNA:tracrRNA duplex [81]. It is tempting to speculate that, in the Type II systems, Cas9-bound tracrRNA provides a scaffold for the crRNA binding and stabilization similarly to Cascade proteins in Type I and Cmr proteins in Type III systems [24–26, 40, 58, 61]. Second, a ternary Cas9–crRNA–tracrRNA complex, using a mechanism that yet has to be defined, locates and binds to a protospacer sequence within the double-stranded DNA in a PAM-dependent process. The absolute requirement of PAM for dsDNA binding by the Cas9t complex implies that PAM serves as a priming site for strand separation or is essential for stabilization of the R-loop structure because dsDNA lacking PAM is not bound. The Cas9t binding to the target sequence in the dsDNA presumably results in an R-loop structure, where one DNA strand is displaced and the complementary strand is paired with the crRNA. PAM is located downstream of the protospacer and differs between different systems. For *S. pyogenes* it is NGG, and for *S. thermophilus* CRISPR1 and CRISPR3 systems, NAAGW and NGGNG, respectively. The PAM is required only for a double-stranded but not a single-stranded DNA binding and cleavage by Cas9t [82, 83]. Third, in the presence of Mg^2+^ ions, DNA is cleaved in both strands within a protospacer 3 nt upstream of the PAM sequence to generate blunt DNA ends. RuvC- and HNH-active sites of Cas9 act on the opposite DNA strands. Taken together, the data demonstrate that the Cas9–crRNA–tracrRNA complex functions as an RNA-guided endonuclease where sequence specificity is dictated by the crRNA while Cas9 provides the cleavage machinery. This establishes a molecular basis for CRISPR-mediated immunity in Type II systems, which solely rely on the signature Cas9 protein.

The simple modular organization of the Cas9t complex paves the way for the engineering of universal RNA-guided DNA endonucleases. While the proof of principle for re-programmable RNA-guided endonucleases have been provided in the pioneering publications [82, 83], recent studies demonstrate that Cas9t can be employed for a precise editing of the human [87–90], mouse [89, 91], zebrafish [92, 93], yeast [94], and bacteria [84] genomes. Furthermore, a catalytically-deficient Cas9 variant was used in bacteria as a tool to specifically repress transcription through the crRNA binding [95]. Moreover, it has been reported [96] that in *Francisella novicida*, a Cas9 variant is involved in regulation of the bacterial gene contributing to the virulence by triggering proinflammatory innate immune response of the eukaryotic host. This finding may open the way for novel Cas9 applications.

## DNA interference in Type III CRISPR–Cas systems

Type III systems are more widespread in archaea that typically encode multiple CRISPR–Cas loci belonging to different subtypes [13–20]. *Pyrococcus furiosus,* for example, contains three Cas protein modules (subtypes I-A, I-B, and III-B) and seven CRISPR arrays [24, 97]. The archaeon *S. solfataricus* holds five Cas protein modules (two belonging to subtype III-B and three—subtype I-A) and six CRISPR arrays, respectively [26]. The Type III CRISPR immune system, however, is not a hallmark of archaeal species. Lactic acid bacterium *S. thermophilus* DGCC7710 accommodates four different CRISPR–Cas loci in the chromosome including the III-B (Online Resource Fig. S2) [31]. Other bacteria, including important pathogens such as mycobacteria and staphylococci, also harbor Type III systems [98].

The Type III CRISPR–Cas systems encode a signature Cas10 protein that bears palm-domain polymerase-specific sequence motifs. Multiple RAMP-family proteins, which are built around a ferredoxin-like fold and are often involved in RNA-binding, are present in the Type III systems [9, 27, 99–101]. Type III systems are further classified into III-A and III-B subtypes. Most of our knowledge on the III-A subtype comes from the *Staphylococcus epidermidis* model system, while *S. solfataricus* and *P. furiosus,* have been used as model systems for III-B (Online Resource Fig. S1). Intriguingly, two different Type III systems seem to target different nucleic acids. In Type III-B systems of *S. solfataricus* and *P. furiosus*, the Cas RAMP module (Cmr) and the crRNA complex recognize and cleave synthetic RNA in vitro [24, 26], whereas the Type III-A system of *S. epidermidis* targets DNA in vivo (Table 4) [98]. On the other hand, the III-B system from *S. islandicus* interferes with plasmid DNA transformation via transcription-dependent DNA targeting and relies on the direct protospacer transcription into RNA [102].

**Table 4:** Features of the Type III CRISPR–Cas systems and effector complexes

Organism	Sub-type	Complex acronym	Repeat length, nt	Repeat sequence	Spacer length, nt	crRNA length, nt	Target	Cleavage position	Complex composition	Ref.
*Staphylococcus epidermidis* RP62A^a^	III-A		36	5′-GTTCTCGTCCCCTTTTCTT CGGGGTGGGTATCGATCC-3′^b^	34–35	37 or 43	dsDNA^c^	nd	nd	[98, 105]
*Pyrococcus furiosus*	III-B	Pf-Cmr	30	5′-GTTCCAATAAGACTA CAAAAGAATTGAAAG-3′	36–42	39 or 45	ssRNA	14 nt from 3′ crRNA end	Cmr1, Cas10, Cmr3–6	[24, 106]
*Sulfolobus solfataricus P2*	III-B	Ss-Cmr	24–25	5′-GATAATCTCTTATAG AATTGAAAG-3′	36–44	45–50	ssRNA (including crRNA)	At UA dinucleotide	Cmr1, Cas10, Cmr3–7	[26]

^a^The effector complex has not been isolated or characterized

^b^Palindromic sequences in the repeat are underlined

^c^According to in vivo data [98]

Unlike repeat sequences in Type I systems, the III-B subtype repeats are non-palindromic [12]. Nevertheless, pre-crRNA processing in the Type III systems is performed by the Cas6 endoribonuclease that contains a conserved ferredoxin-like fold, but, unlike the Cas6 of the Type I system, uses a different protein surface to interact with a non-palindromic repeat sequence. Processing of crRNA in *P. furiosus* occurs in two steps. Firstly, Cas6 of *P. furiosus* cuts pre-crRNA within a repeat region to yield a 66- to 72-nt 1× intermediate [22, 103, 104]. Secondly, subsequent degradation of the 3′-end of the 1× intermediate by unknown nucleases yields two crRNA populations, 45 and 39 nt in length, that both contain a conserved 8-nt 5′-handle originating from the repeat region but trimmed at 3′-end. Accordingly, mature crRNA contains 37 and 31 nt belonging to the spacer region [24].

The repeat sequence in the III-A subtype, as exemplified by *S. epidermidis,* is partially palindromic [105]. Pre-crRNA processing in vivo requires Cas6, Csm4, and/or Cas10 proteins and cleavage occurs at the base of the hairpin stem [105]. The cleavage pattern of pre-crRNA presumably follows a mechanism similar to that of Cas6 endoribonuclease of Type I-E and I-F systems [41, 42, 57], and Se-Cas6 most likely is involved in pre-crRNA processing in *S. epidermidis*. Further maturation of the 1× intermediate proceeds via 3′-end trimming to yield 43- and 37-nt crRNAs and requires Csm2, Csm3, and Csm5 proteins [105].

The mechanism of invading nucleic acid degradation has been studied in vitro for the Type III-B systems of *P. furiosus* and *S. solfataricus,* and in vivo for the III-A system of *S. epidermidis*. Effector complexes of *P. furiosus* and *S. solfataricus* (Pf-Cmr and Ss-Cmr complexes) have been purified and biochemically characterized. Unlike other CRISPR–Cas systems that target DNA, both III-B effector complexes target single-stranded RNA (Table 4) [24, 26, 106].

### *P. furiosus*

Effector complex of *P. furiosus* (Pf-Cmr) consists of six Cas proteins (Cas10, Cmr1, and Cmr3–6) and crRNA that originates from seven distinct spacer-repeat arrays. The Pf-Cmr complex cleaves complementary target RNA in vitro at the fixed 14 nt distance from the 3′-end of the crRNAs (Table 4). Because mature crRNAs of *P. furiosus* are of two different lengths, 39 and 45 nt, respectively, the target RNA is cleaved at two distinct positions, implying that the Pf-Cmr complex uses a molecular ruler mechanism [24]. The active Pf-Cmr complex can be assembled in vitro by mixing six individual recombinant Cas proteins with 39- or 45-nt crRNA. The 8-nt 5′-handle of crRNA is critical for Pf-Cmr complex activity, and synthetic crRNAs bearing 5′-handle can be used to direct cleavage of novel targets [106]. On the other hand, biochemical analysis reveals that Cmr5 is dispensable for RNA cleavage activity, therefore the candidate protein responsible for RNA cleavage remains to be established [24]. Evidence that supports in vivo functionality of this system has recently been reported [106].

The hallmark Cas10 protein of Type III systems is a multidomain protein that has a permutated HD superfamily nuclease domain at the N-terminus, followed by a zinc finger and polymerase-like domains [27]. HD-domain is important for DNA degradation by the Cas3 protein in the Type I systems; however, it seems to be dispensable for Pf-Cmr-mediated RNA cleavage in vitro [107]. Crystal structure of the truncated Cas10 version from *P. furiosus* lacking the HD domain (Cas10 ΔHD) shows structural similarities to an adenylyl-cyclase rather than a polymerase. Moreover, mutations of the conserved ATP and divalent metal ion binding residues have no effect on RNA cleavage activity of the Pf-Cmr complex [107]. It has been shown recently that Cas10 interacts with the Cmr3 protein that shares similarity to Cas6e and Cas6f proteins [109, 110]. Thus, it is likely that Cas10 plays a structural rather than a catalytic role in the Pf-Cmr complex [107, 108].

### *S. sulfolobus*

The Cmr complex purified from *S. sulfolobus* (Ss-Cmr) consists of seven Cas proteins (Cmr1, Cas10, and Cmr3–7) and crRNA of various length (Table 4). Interestingly, the Cmr7 protein is present only in *S. solfataricus* species. In contrast to the Pf-Cmr complex that exploits a molecular ruler mechanism for the target RNA cleavage, the Ss-Cmr complex cleaves RNA at UA dinucleotides in a sequence-specific manner. Moreover, the Ss-Cmr complex cleaves both target and guide RNA (crRNA) sequences in vitro, although a single crRNA molecule supports the degradation of multiple RNA targets [26]. The electron microscopy structure of the Ss-Cmr complex and the sub-complex including only Cas10, Cmr3, and Cmr7 proteins shows a clamp-like structure containing a deep cleft, which could accommodate double-stranded RNA [26].

### *S. epidermidis*

*S. epidermidis* RP62A strain harbors a single III-A CRISPR locus containing nine *cas* genes flanked by three repeat-spacer units. The targeting mechanism in the *S. epidermidis* model system is not fully understood and is limited to in vivo studies. Furthermore, the effector complex composition remains to be established. Nevertheless, in vivo studies reveal two important clues: (1) in contrast to the III-B systems, the protospacer DNA, rather than corresponding mRNA is targeted by the III-A CRISPR–Cas systems, and (2) III-A systems are PAM-independent and prevention of autoimmunity is achieved by checking the complementarity between the crRNA 5′-handle and the 3′-flanking sequence in the vicinity of the protospacer. The perfect match that will occur in the repeat region specifies self DNA while a mismatch tags alien DNA and triggers destruction [111]. However, it remains to be demonstrated that DNA target is cleaved in vitro by the Type III-A effector complex.

### Mechanism of DNA-interference in the Type III systems

**Fig. 4:**
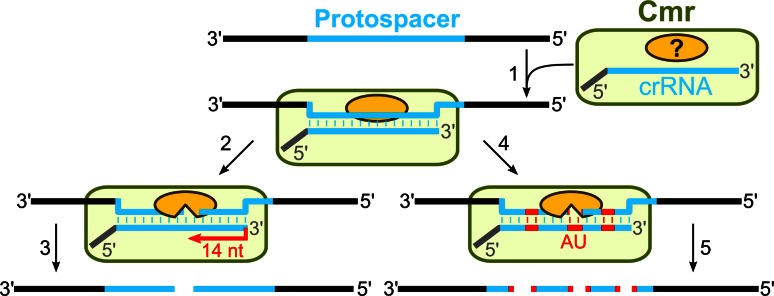
Interference in the Type III CRISPR–Cas systems. In the case of the Type III-B CRISPR–Cas system, the Cmr complex scans RNA and crRNA basepairs with a matching protospacer sequence (*1*). Two different RNA cleavage mechanisms are proposed. The Cmr complex from *P. furiosus* exploits the ruler mechanism to introduce cuts in the target RNA 14 nt from the 3′-end of crRNA (*2*) to yield two product fragments (*3*). The Cmr complex of *S. sulfolobus* guided by crRNA cuts the target RNA in a sequence-specific manner at UA dinucleotides (*4*) at multiple positions (*5*). The Cmr complex components involved in the cleavage have yet to be established

In summary, genetic and biochemical studies of the CRISPR-encoded immunity in the Type III CRISPR–Cas systems are consistent with the following general mechanism of interference (Fig. 4). Firstly, the CRISPR repeat region is transcribed into a long primary pre-crRNA which undergoes a two-step processing to yield mature crRNA of two different lengths which contain an 8-nt 5′-handle originating from the repeat sequence and trimmed spacer 3′-end. Despite the differences in repeat sequences (partially palindromic vs. non-palindromic in the III-A and III-B systems, respectively), Cas6 ribonuclease contributes to the primary processing stage. Secondly, the mature crRNA in the Type III-B system is incorporated into an effector complex which targets RNA in vitro using crRNA as a guide. The complex composition, stoichiometry, and mechanisms of the target RNA degradation in vitro differ between *P. furiosus* and *S. solfataricus* complexes. The nucleases involved in the target RNA (Type III-B) or DNA (Type III-A) cleavage remain to be established. Thirdly, unlike the effector complexes of Type I and Type II systems, the effector complexes of III-A and III-B systems achieve interference in a PAM-independent manner.

## Concluding remarks

CRISPR–Cas systems provide acquired immunity against viruses and plasmids and come in different flavors. Despite the differences, the silencing of the invading nucleic acid (DNA for Type I and II, RNA for Type III-B) includes an obligatory cleavage step, and in all cases crRNA is used as a guide to address the cleavage machinery to the target site. The effector complex that binds crRNA and triggers cleavage differs strikingly between different CRISPR subtypes. In most of the Type I systems studied to date, crRNAs are incorporated into a multisubunit effector complex called Cascade (CRISPR-associated complex for antiviral defence), which binds to the target DNA and triggers degradation by a signature Cas3 protein. The composition of the Cascade differs between subtypes, but in all cases it is arranged as a multisubunit aggregate that provides a scaffold for crRNA binding. In the Type II systems, the effector complex functions as an RNA-guided endonuclease where sequence specificity is dictated by the crRNA while Cas9 provides the cleavage machinery. It is likely that, in the Type II systems, tracrRNA provides a scaffold for the crRNA binding. In Type III-B CRISPR–Cas systems of *S. solfataricus* and *P. furiosus,* the Cas RAMP module (Cmr) and crRNA effector complexes recognize and cleave synthetic RNA in vitro. In the Type I systems, the Cas3 helicase/nuclease acts as a slicer that degrades invading foreign DNA. In the Type II systems, Cas9 protein cuts both DNA strands within a protospacer using two different active sites that act on opposite DNA strands. Despite recent progress in deciphering mechanisms of CRISPR-mediated immunity, it remains to be established: (1) how interference is achieved in the Type III-A CRISPR–Cas systems, (2) how the effector complex locates the target site in the invading nucleic acid, (3) how supercoiling contributes to the target site location/cleavage in the CRISPR systems acting on DNA, and (4) how PAM is recognized. The answers to these and other questions will contribute to further understanding of the molecular mechanisms of CRISPR-encoded immunity, and will open new avenues for future applications of CRISPR systems in biotechnology.

## Electronic supplementary material

Below is the link to the electronic supplementary material.

Supplementary material (PDF 1192 kb)

## Abbreviations

Cas: CRISPR-associated
Cascade: CRISPR-associated complex for antiviral defense
CRISPR: Clustered regularly interspaced short palindromic repeats
crRNA: CRISPR RNA
ds: Double-stranded
nt: Nucleotide
PAM: Protospacer-adjacent motif
RAMP: Repeat associated mysterious protein
RNP: Ribonucleoprotein
ss: Single-stranded
tracrRNA: Trans-activating CRISPR RNA
WT: Wild-type
