# Supplementary material for: Molecular mechanisms of CRISPR-mediated microbial immunity
Source: Cell Mol Life Sci. 2013 Aug 20;71(3):449–65. doi: 10.1007/s00018-013-1438-6 (PMC3890593; doi:10.1007/s00018-013-1438-6)
Supplement: Supplementary file 1 — Supplementary material (PDF 1192 kb) [file 18_2013_1438_MOESM1_ESM.pdf]

# Supplementary Figures

## Molecular mechanisms of CRISPR-mediated microbial immunity

Giedrius Gasiunas[1], Tomas Sinkunas[1], and Virginijus Siksnys[1*]

[1]*Institute of Biotechnology, Vilnius University, Graiciuno 8, LT-02241, Vilnius, Lithuania*

[*] Corresponding author

Mail: Institute of Biotechnology, Graiciuno 8, Vilnius LT-02241, Lithuania

Tel: +370-5-2602108; Fax: +370-5-2602116; E-mail: siksnys@ibt.lt

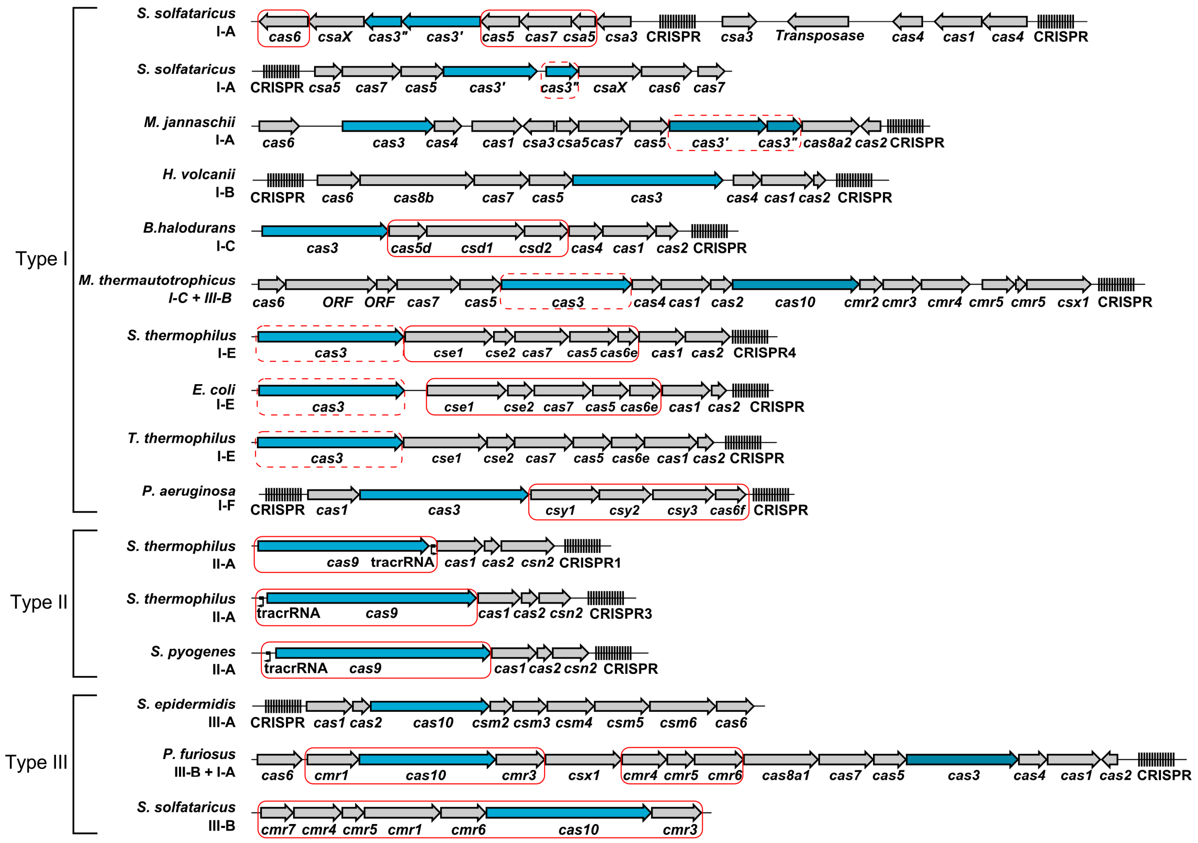


**Fig. S1** CRISPR-Cas systems for which effector complexes or their components were characterised *in vitro* or/and *in vivo*. Genes encoding proteins of effector complexes are boxed. Genes encoding biochemically characterised Cas3 proteins are enclosed in dashed boxes. Signature genes of CRISPR-Cas systems are coloured in blue.

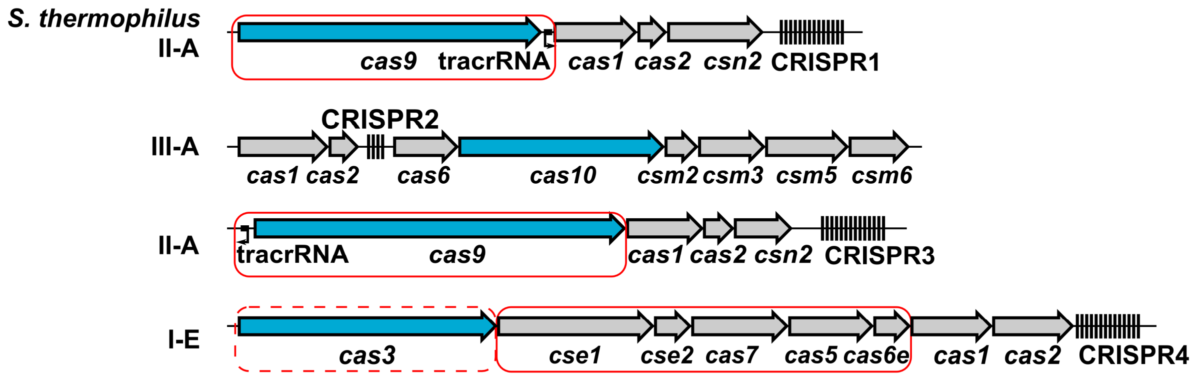


**Fig. S2** CRISPR-Cas systems of the *S. thermophilus* DGCC7710 strain. Cas genes encoding effector complex proteins are boxed. A gene for an accessory Cas3 protein of Type I system that acts as a slicer degrading invading DNA is enclosed in a dashed box. Signature genes of each subtype CRISPR-Cas are coloured in blue.
